# Engineered compact pan-neuronal promoter from Alphaherpesvirus LAP2 enhances target gene expression in the mouse brain and reduces tropism in the liver

**DOI:** 10.1038/s41434-023-00430-0

**Published:** 2023-11-27

**Authors:** Carola J. Maturana

**Affiliations:** Princeton Neuroscience Institute, Princeton University, Princeton, NJ USA

**Keywords:** Cellular neuroscience, Neurological disorders, Genetic engineering, Genetic vectors, Gene regulation

## Abstract

Small promoters capable of driving potent neuron-restricted gene expression are required to support successful brain circuitry and clinical gene therapy studies. However, converting large promoters into functional MiniPromoters, which can be used in vectors with limited capacity, remains challenging. In this study, we describe the generation of a novel version of alphaherpesvirus latency-associated promoter 2 (LAP2), which facilitates precise transgene expression exclusively in the neurons of the mouse brain while minimizing undesired targeting in peripheral tissues. Additionally, we aimed to create a compact neural promoter to facilitate packaging of larger transgenes. Our results revealed that MiniLAP2 (278 bp) drives potent transgene expression in all neurons in the mouse brain, with little to no expression in glial cells. In contrast to the native promoter, MiniLAP2 reduced tropism in the spinal cord and liver. No expression was detected in the kidney or skeletal muscle. In summary, we developed a minimal pan-neuronal promoter that drives specific and robust transgene expression in the mouse brain when delivered intravenously via AAV-PHP.eB vector. The use of this novel MiniPromoter may broaden the range of deliverable therapeutics and improve their safety and efficacy by minimizing the potential for off-target effects.

## Introduction

The field of translational neuroscience has brought forth innovative technologies with immense potential for the treatment of prevalent neurological disorders causing disability. Among these advancements, recombinant adeno-associated virus (rAAV)-based gene therapy has emerged as a highly promising technology [1]. rAAV is extensively employed in both in vitro and in vivo studies for neural circuit mapping because of its exceptional transfection efficiency and ability to maintain the stable expression of transgenes [2]. However, targeting transgene expression in specific tissues and cell types remains challenging [3, 4]. The use of a cell type-specific promoter can result in improved targeting, which may limit the detection of transgenic proteins by antigen-presenting cells and eliminate undesirable immune responses [5]. Regrettably, most neuron-specific promoters exhibit modest levels of transgene expression [6]. As a result, substantial doses of AAV vectors are frequently administered to achieve therapeutic effectiveness. However, this approach can give rise to significant drawbacks, including thrombotic microangiopathy (TMA) and hepatotoxicity, as an adverse effect [7, 8]. Promoters with constitutive activity, such as human cytomegalovirus immediate early enhancer (CMV; 752 bp) [9], calcium/calmodulin-dependent protein kinase II alpha (CaMKIIa; 1,300 bp) [10], CMV enhancer fused to the chicken beta-actin promoter (CAG; 912 bp) [11, 12] and neuron-specific enolase (NSE; 1800 bp) [13], have demonstrated remarkable potency in selectively driving transgene expression in neurons. However, the substantial size of these promoter sequences restricts the use of large therapeutic transgenes or the incorporation of multiple small transgenes. Many strategies have been devised to enhance cell-specific expression and maximize AAV payload capacity. These strategies include the utilization of dual or triple AAV systems to divide large transgenes into multiple segments [14]; design of therapeutic mini-genes [15]; and/or incorporation, or deletion of viral regulatory elements and transcription factor binding sites (TFBSs) or enhancers [16–18]. Prior studies have highlighted the effectiveness of inserting TFBSs directly upstream of native promoters. This not only amplifies promoter activity but also substantially augments the specificity of transgene targeting [19]. Likewise, enhancers, those DNA regulatory components responsible for conferring cell type-specific gene expression, when paired with a small promoter, enable accurate tagging of distinct brain regions and cell types, transcending various species [20]. Notably, certain enhancers like the mDlx enhancer retain their specificity even when introduced intravenously into mice through dedicated AAV-PHP.eB vectors [21]. Cutting-edge genomic techniques, such as open chromatin analysis, have played a pivotal role in the discovery of new enhancers, thereby expanding available toolkits [22–26]. Nevertheless, ongoing endeavors to identify and validate enhancers, particularly those exhibiting enhanced efficiency after systemic delivery, will further augment the capabilities of AAV-based tools in the realm of neuroscience research.

The swine herpesvirus pathogen pseudorabies virus (PrV) has been extensively used in CNS neuronal network analyses because it can infect all types of neurons [27, 28]. The constitutive PrV alphaherpesvirus latency-associated promoter (LAP) has a cluster of two tandem promoter sequences (LAP1 and LAP2) that drive sustained transcription of latency-associated transcripts (LATs) [29–32]. In our previous studies, we demonstrated that systemic administration of PrV LAPs drives potent neuron-specific transgene expression independent of PrV infection in the brain and spinal cord, with an activity profile of LAP2 > LAP1_2 tandem» LAP1 [33]. Here, we modified LAP2 to restrict transgene expression in the brain, reduce its distribution to peripheral tissues, and maximize the carrying capacity of AAV. The newly engineered 278 bp promoter that drives an mCherry reporter (MiniLAP2-mCherry) was delivered intravenously into adult mice via AAV-PHP.eB vector at a dose of 5 × 10^11^ genome copies (gc)/mouse. PHP.eB-LAP2-mCherry was similarly injected to facilitate a side-by-side comparison. After four weeks, we examined mCherry expression in the brain, spinal cord, liver, kidney, and skeletal muscle of these mice. We found that the PHP.eB-MiniLAP2 vector induced specific and potent transgene expression in neuronal cells of the mouse brain. In contrast, minimal transgene expression was detected in the spinal cord and liver but not in the kidney and skeletal muscles. These findings suggest that fine-tuning promoter-mediated tissue specificity may be accomplished by adjusting promoter design and incorporating selected TFBSs. In addition to improved specificity, the compact nature of this promoter has proven to be particularly advantageous for scAAV vectors. Hence, the truncated version of LAP2 facilitates the packaging of large or multiple small transgenes without compromising expression efficiency, thereby expanding therapeutic options for addressing brain disorders.

## Results and discussion

**Fig. 1:**
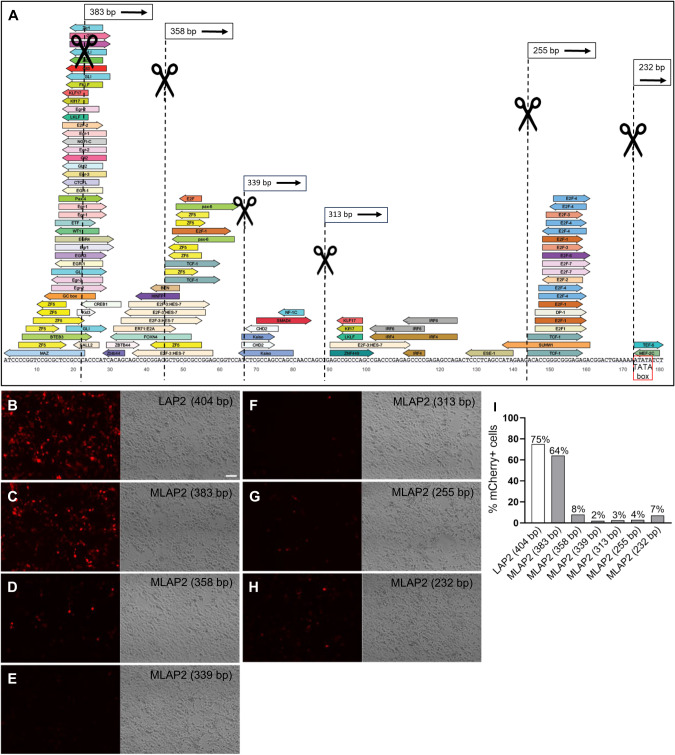
Bioinformatic design of six MiniPromoters targeting neuron specific. **A** Prediction of potential transcription factor binding sites (TFBSs) at the 5’ end of the native LAP2 sequence using TRANSFAC®. The identification of elements potentially regulating neuron expression will allow us to design a series of 5’ deletions (21, 46, 65, 91, 149, and 172 bp) immediately upstream of the TATA box (red box), resulting in six promoter variants (black box). The MiniPromoter variant sizes were 383, 358, 339, 313, 255, and 232 bp. Native mCherry expression in 293 T cells is shown at 3 days post transfection with **B** LAP2 full-length 404 bp, **C** MiniLAP2 variant 383 bp, **D** MiniLAP2 variant 358 bp, **E** MiniLAP2 variant 339 bp, **F** MiniLAP2 variant 313 bp, **G** MiniLAP2 variant 255 bp, **H** MiniLAP2 variant 232 bp plasmid. Scale bars, 100 µm. **I** Percentage of 293 T cells expressing mCherry following transfection with LAP2 or various MiniLAP2 variant plasmids at the 3-day time point. MLAP2, MiniLAP2 variant.

Considering the critical role of the 3’ end region in promoter-mediated gene transcription [34], we postulated that the LAP2 sequence could potentially be minimized by removing specific DNA elements and motifs positioned directly upstream of the TATA box. We used TRANSFAC® and JASPAR software to predict potential TFBSs within the LAP2 sequence. Thus, we introduced a series of 5’ truncations (21, 46, 65, 91, 149, and 172 bp) immediately upstream of the TATA box, depending on the location of critical TFBSs for expression in neurons, resulting in six promoter variants (Fig. 1A). To assess and compare promoter activities, MiniLAP2 variants were used in constructs designed to drive expression of the fluorescent protein mCherry. Both native and truncated promoter plasmids were transfected into human embryonic kidney 293 (293 T) cells, which exhibit properties similar to those of immature neurons [35]. We evaluated the transfection efficiency based on mCherry fluorescence intensity. Significantly elevated levels of mCherry fluorescence were noted in cells transfected with the truncated 383 bp construct (64%), mirroring the expression levels observed in cells transfected with the native LAP2 construct (404 bp) (75%) (Fig. 1B, C, I). In contrast, none of the other five MiniLAP2 constructs exhibited notable mCherry expression in these cells, indicating a substantial reduction in promoter function (<10%) (Fig. 1D–H, I). These findings suggest that promoter sequence elements located between positions 21 and 172 bp relative to the TATA box play a critical role in facilitating efficient transcription.

**Fig. 2:**
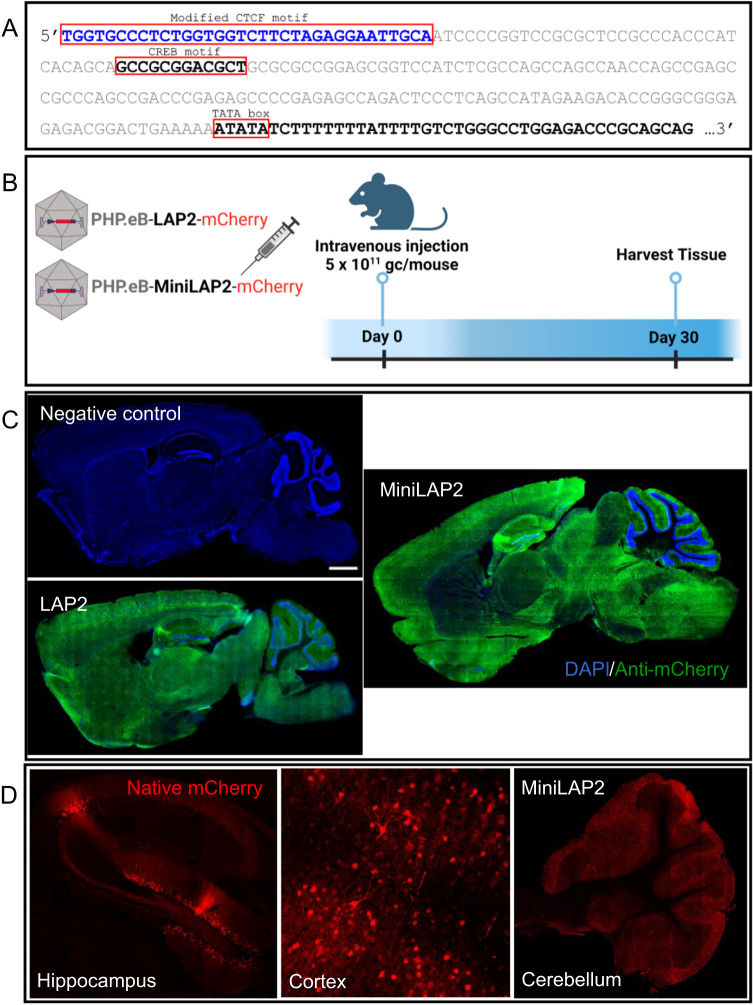
MiniLAP2 and LAP2 drive transgene expression in mouse brains with equivalent efficiencies. **A** The LAP2 sequence was modified upstream (5’ end) of the transcriptional start signal (as indicated by the TATA box). The first 160 base pairs (bps) of LAP2 were removed (as shown in grey), although the CREB-binding motif was preserved. A modified CTCF motif (blue) is added immediately upstream of the CREB site. **B** LAP2 (404 bp) and MiniLAP2 (278 bp) promoters were individually packaged into ssAAV-PHP.eB vector to drive the transcription of mCherry fluorescent reporter. Packaged AAV vectors were administered by unilateral intravenous injection into the retro-orbital sinus at a dose of 5 × 10^11^ gc per mouse. Negative control mice were inoculated with buffer without AAV. The brain, spinal cord, liver, kidney, and quadriceps muscles were collected 30 days after inoculation. **C** Representative mCherry immunofluorescence images of sagittal brain sections documenting transgene expression driven by both LAP2 and MiniLAP2, with no expression observed in negative control mice. Nuclei were counterstained with DAPI (blue). **D** Representative confocal images show native mCherry immunofluorescence (red) from MiniLAP2 in hippocampus, cortex, and cerebellum. Scale bar, 1 mm.

A comprehensive analysis of the potential TFBSs in this critical area revealed only a single CREB motif. Previous studies of other members of the herpesvirus family, including herpes simplex virus type 1 (HSV-1), revealed that CREB motifs located proximal to the TATA box are necessary to support basal neuronal gene expression [36]. Based on the overall architectural similarity between the LAP2 and HSV-1 gene promoters, we designed a minimal LAP2 promoter by removing the first 160 bp immediately upstream of the TATA box while retaining the 12 bp CREB motif (Fig. 2A). Given that attempts to minimize the promoter could potentially compromise specificity and weaken transgene expression, leading to increased susceptibility to gene silencing, we implemented a strategy to address this issue. We inserted a modified CTCF motif directly upstream of the CREB site to use it as an enhancer-blocking insulator (Fig. 2A). This approach was intended to prevent unwanted interactions and maintain promoter integrity, thereby enhancing transgene expression while preserving specificity. CTCFs contribute to a diverse array of regulatory functions, including transcriptional activation and repression, genome instability, RNA splicing, RNA binding, and enhancer/promoter insulation [37]. For instance, Zimmerman et al. [19], demonstrated that the orientation of the CTCF motif and/or the number of accessible zinc-finger binding sites may have an impact on transgene expression. These effects were observed when the CTCF element was inserted upstream or downstream of the CMV or elongation factor-1 alpha (EF1α) promoter. More specifically, the authors discovered that incorporating a modified CTCF motif (34 bp) with an upstream stabilizer sequence, which had the ability to bind eight of the eleven zinc fingers of its CCCTC-binding factor partner, resulted in substantial enhancement of transgene expression compared to the native motif. This improvement was observed when the modified CTCF motif was inserted downstream of the EF1α promoter [19].

**Fig. 3:**
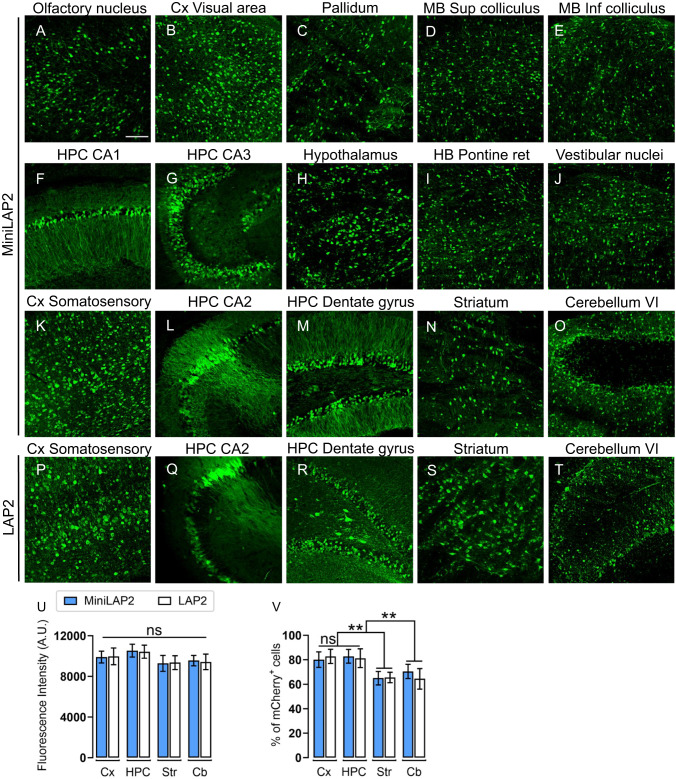
MiniLAP2 drives widespread and potent transgene expression in all regions of the mouse brain. Representative confocal images documenting anti-mCherry signals (green) in the brain tissue of mice 30 days after inoculation with PHP.eB-MiniLAP2, including **A** anterior olfactory nucleus, **B** Cx visual area, **C** pallidum, **D** superior colliculus of the midbrain (MB), **E** inferior colliculus of the MB, **F** HPC CA1, **G** HPC CA3, **H** hypothalamus, **I** hindbrain (HB) pontine reticular nuclei, **J** vestibular nuclei of the medulla oblongata, **K** Cx somatosensory, **L** HPC CA2, **M** HPC dentate gyrus, **N** striatum, and **O** cerebellum VI. Representative confocal images of PHP.eB-LAP2 mediated mCherry expression (green) in the **P** Cx somatosensory, **Q** HPC CA2, **R** HPC dentate gyrus, **S** striatum, and **T** cerebellum VI. All images are stacked confocal sections (scale bar, 100 µm). **U** Quantification of the fluorescence intensity of the mCherry signal driven by PHP.eB-MiniLAP2 and PHP.eB-LAP2 in the cortex (Cx), hippocampus (HPC), striatum (Str), and cerebellum (Cb). **V** Quantification of the percentage of mCherry-positive cells after inoculation with AAV-MiniLAP2 or AAV-LAP2 in the Cx, HPC, Str, and Cb. Data are represented as the mean ± SEM; *n* = 4 animals per group; three tissue sections were analyzed for each animal. Statistical significance was determined by Student’s *t*-test, with a *P*-value < 0.05 considered to be statistically significant. **P < 0.002; ns, not significant.

To characterize the novel MiniLAP2 promoter (278 bp) and explore its capacity to drive transgene expression in vivo, we used engineered ssAAV-PHP.eB capsids for gene transfer. This capsid can cross the blood-brain barrier and promote efficient biodistribution and transduction in the CNS [33, 38, 39]. Packaged PHP.eB-MiniLAP2-mCherry and PHP.eB-LAP2-mCherry were delivered by unilateral injection of 5 ×10^11^ gc/mouse into the retro-orbital sinus. The brain, spinal cord, liver, kidney, and quadriceps muscle were collected 30 days post-inoculation to evaluate and compare reporter gene expression (Fig. 2B). A validated anti-mCherry antibody [33] was used to amplify the reporter protein signal. Representative images of sagittal brain sections revealed widespread and strong mCherry expression in both the promoters (Fig. 2C). Moreover, we detected abundant native mCherry signal in the CA2 region and dentate gyrus of the hippocampus, as well as in the cortex and cerebellum (Fig. 2D). Confocal microscopy demonstrated that the expression of the transgene driven by PHP.eB-MiniLAP2 was remarkably enriched in various regions of the brain (Fig. 3A–O). Furthermore, when measuring fluorescence intensity (Fig. 3U), there were no statistically significant differences between the two promoters in the cortex, hippocampus, striatum, or cerebellum (*P* < 0.05) (Fig. 3K–O, P–T). Similarly, no discernible distinctions were observed when quantifying the number of mCherry-positive cells (Fig. 3V). Specifically, our findings revealed that the percentages of mCherry-positive cells identified in the cortex and hippocampus (80–83%, *P* < 0.002) were significantly higher than those detected in the striatum and cerebellum (64–70%), regardless of the promoter construct used (Fig. 3V). These findings established that both promoters drive analogous transgene expression within the brain following a single systemic AAV administration, even in the presence of a 40% reduction compared to the native sequence.

**Fig. 4:**
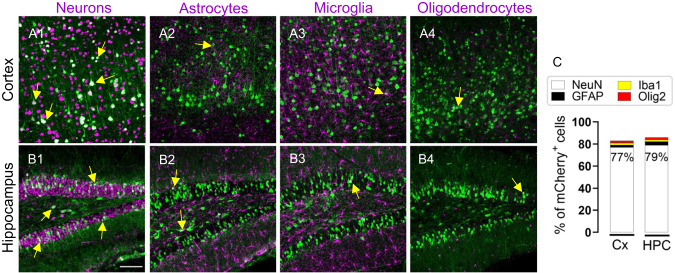
PHP.eB-MiniLAP2 transgene expression was predominantly detected in neurons. **A**, **B** Representative confocal images of sagittal brain sections from mice 30 days after inoculation with PHP.eB-MiniLAP2, and immunostained with mCherry antibody (green). The staining patterns of (**A1, B1**) neurons (detected by staining with anti-NeuN, magenta), (**A2, B2**) astrocytes (detected by staining with anti-GFAP, magenta), (**A3, B3**) microglia (detected by staining with anti-Iba1, magenta), and (**A4, B4**) oligodendrocytes (detected by staining with anti-Olig2, magenta) in the cortex (Cx) and hippocampus (HPC). Arrows denote the merging of both channels (white). While NeuN, GFAP, and Iba1 signals can be detected both within the cell nucleus and cytoplasm, Olig2 signal is limited primarily to the nucleus (scale bar, 100 µm). **C** Percentage of transgene-expressing neurons, astrocytes, microglia, and oligodendrocytes in the Cx and HPC. Images are stacked confocal sections; *n* = 4 per group; three tissue sections were analyzed per animal.

To determine whether MiniLAP2 drives neuron-specific expression in vivo, we performed co-immunostaining of mCherry protein with neuronal markers (anti-neuronal nuclear protein [NeuN]) and non-neuronal cell types, including astrocytes (anti-glial fibrillary acidic protein [GFAP]), microglia (anti-ionized calcium-binding adapter molecule 1 [Iba1]), and oligodendrocytes (anti-oligodendrocyte transcription factor 2 [Olig2]) (Fig. 4). Within the cortex, mCherry was detected in large pyramidal neurons in somatomotor area layers II–III and V (Fig. 4A1, C). Prominent expression was also detected in the hippocampus, specifically in neurons of the granular layer and hilus of the dentate gyrus (Fig. 4B1, C). Conversely, very few mCherry-positive non-neuronal cells were detected, including only 1% and 3% of astrocytes in cortex and hippocampus, respectively. Similarly, only 1% microglia and 1% oligodendrocytes were mCherry-positive in mice inoculated with the MiniPromoter (Fig. 4A2–A, B2–B4, C). These findings indicated that MiniLAP2 drives neuron-specific transgene expression in the brain of mice systemically inoculated with AAV-PHP.eB.

**Fig. 5:**
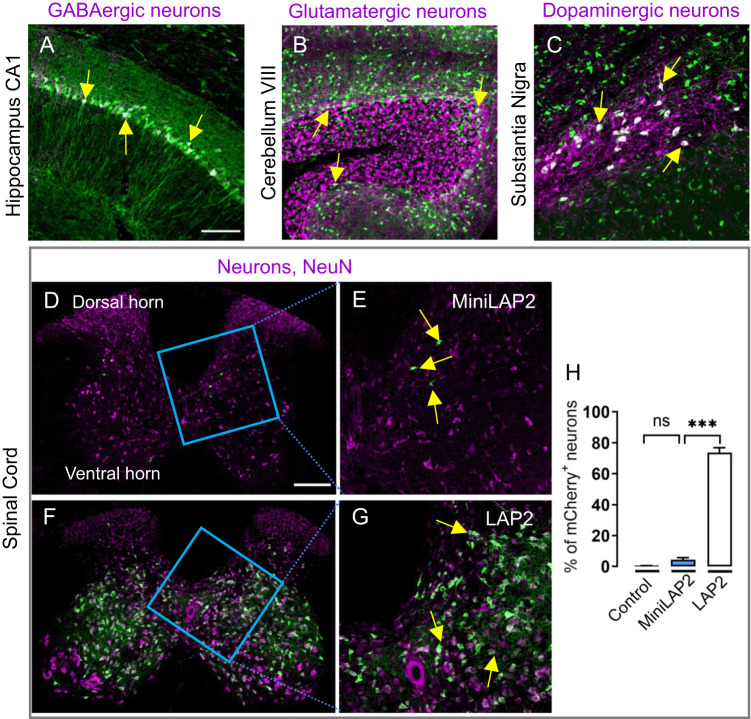
MiniLAP2 drives transgene expression in GABAergic, glutamatergic, and dopaminergic neurons. Representative confocal images of sagittal brain sections from mice immunostained with mCherry antibody (green) and **A** anti-GAD67 (magenta) to detect GABAergic neurons in the hippocampus CA1, **B** anti-vGLUT2 (magenta) to detect glutamatergic neurons in the cerebellum VIII, and **C** anti-TH (magenta) to detect dopaminergic neurons in the substantia nigra. Representative confocal image of a cross section of the lumbar spinal cord immunostained with mCherry antibody (green) and anti-NeuN (magenta) are shown for **D**, **E** AAV-MiniLAP2 and **F**, **G** AAV-LAP2. **E**, **G** A higher-magnification image of the dorsal horn reveals minimal transgene expression. Arrows denote the merging of both channels (white). Scale bars: 100 and 200 µm. **F** Percentage of cells expressing mCherry driven by negative control, AAV-LAP2 and AAV-MiniLAP2. Data are reported as mean ± SEM; *n* = 4 animals per group; three tissue sections were analyzed for each animal); ***P < 0.001; ns, not significant by one-way ANOVA with Tukey’s post-hoc test.

We next evaluated mCherry expression in regions containing specific inhibitory and excitatory neurons and performed immunostaining to determine whether mCherry was preferentially expressed in GABAergic (anti-glutamic acid decarboxylase [GAD67]-positive), glutamatergic (anti-vesicular glutamate transporter 2 [vGlut2]-positive), or dopaminergic (anti-tyrosine hydroxylase [TH]-positive) cells. Representative confocal images revealed that GAD67 was concentrated primarily in the soma of GABAergic neurons, as well as in an overlapping pattern with mCherry in pyramidal cells in the hippocampal CA1 region (Fig. 5A). We also detected MiniLAP2-driven transgene expression in glutamatergic neurons in the cerebellum (Fig. 5B) and well as those in the substantia nigra (TH-positive neurons) (Fig. 5C). In addition to the brain, we evaluated PHP.eB-MiniLAP2-mediated transgene expression in the spinal cord (Fig. 5D–H). Negligible transgene expression was observed in the dorsal and ventral horns of the lumbar spinal cord, in which only approximately 4% of mCherry-positive cells were co-immunostained with anti-NeuN (Fig. 5D, E, H). This notable 70% reduction, compared to native LAP2 (Fig. 5F–H), strongly suggests that the design of the MiniLAP2 sequence facilitates selective transgene expression specifically in brain neurons while exhibiting limited expression in the spinal cord.

To evaluate the distribution of AAV-MiniLAP2 in the cellular compartments of brain neurons, we examined the images and performed quantitative analysis of the AAV vector genome (DNA) and transcript (mRNA) in mouse brains 30 days after systemic administration of the vector. For these experiments, we used RNAscope/BaseScope® approaches to detect mCherry mRNA and AAV DNA, respectively. In both the cortex and the hippocampal CA2 region, individual dots representing DNA signals were clearly visible within cell nuclei, with positivity rates of 68% and 75%, respectively (Supplementary Fig. 1A–B, 1E, 1I). A semi-quantitative assessment of DNA was performed using the Advanced Cell Diagnostics (ACD) scoring criteria (see Materials and Methods). Using this system, the cortex and hippocampus received an ACD score of 3, corresponding to an average of 10–15 viral gc per cell, and <10% of the dots were localized in clusters (Supplementary Fig. 1J). Interestingly, the signal levels denoting the viral transcripts in these regions were more homogeneous. We found that mCherry RNA transcripts were concentrated as foci in all regions of the cortex that were evaluated (Supplementary Fig. 1D, C) and that transcription was much more active in hippocampal CA neurons than in the dentate gyrus (Supplementary Fig. 1D, F). Transgene transcripts were detected in 71% and 85% of cells in the cortex and hippocampus, respectively (Supplementary Fig. 1K). Notably, while mRNA was detected in both the cytoplasm and nucleus, we cannot rule out the possibility that nuclear location may result (all or in part) from interactions between the antisense probe and the sense strand of AAV DNA. We determined that both regions exhibited >15 dots per cell, with >10% of the dots found in the clusters (Supplementary Fig. 1L). As expected, increased mCherry levels were discerned following the systemic delivery of PHP.eB-MiniLAP2, affirming the operational efficacy of MiniLAP2.

**Fig. 6:**
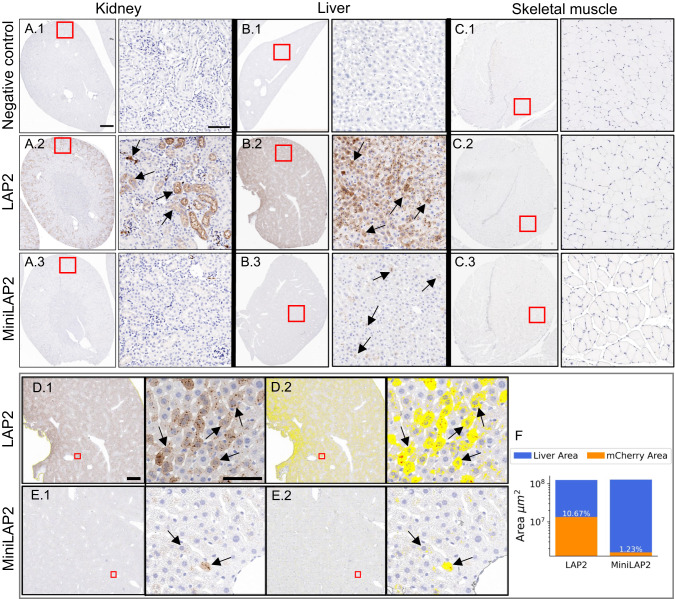
Systemic administration of PHP.eB-LAP2 and PHP.eB-MiniLAP2 leads to differential transgene expression in liver, kidney, and muscle tissues. Representative images of mCherry-positive cells (brown) in **A** the kidney, **B** liver, and **C** skeletal muscle tissue from mice inoculated with (**A1, B1, C1**) negative control, (**A2, B2,** **C2**) PHP.eB-LAP2, or (**A3, B3, C3**) PHP.eB-MiniLAP2. (**D1, E1**) Representative images of mCherry immunostaining and (**D2, E2**) images corresponding to the morphometric analysis used to quantify mCherry immunostaining in liver tissue from mice inoculated with PHP.eB-LAP2 and PHP.eB-MiniLAP2. The inset squares show the fields shown at a higher magnification (on the right). Arrows indicate the immunostained cells. Scale bars, 200 µm (**A**–**C**), 1 mm, and 100 µm (**D**, **E**), as indicated. **F** Percentage of mCherry-positive nuclei per unit area (μm^2^) of liver tissue from mice inoculated with PHP.eB-LAP2 or PHP.eB-MiniLAP2; *n* = 4 animals per group, with two tissue sections analyzed per animal.

We then examined the AAV-MiniLAP2 performance in peripheral tissues, utilizing immunohistochemical and morphometric techniques to assess mCherry expression (Fig. 6). Upon systemic administration of PHP.eB-MiniLAP2, we observed absence of transgene expression in the kidney (Fig. 6A3) and negligible expression of mCherry in the liver (Fig. 6B3) which contrasts markedly with the expression levels observed for the native LAP2 promoter (Fig. 6A2, C2). While no expression was observed for both promoters in skeletal muscles like negative control (Fig 6C1-3). mCherry expression in the liver was quantified as the number of positive pixels per square micron detected within the liver lobule. Using this method, we observed significantly lower mCherry immunostaining in liver tissues from mice inoculated with PHP.eB-MiniLAP2 than in mice inoculated with PHP.eB-LAP2, detected at 1% and 11%, respectively (Fig. 6D–F). Notably, previous studies utilizing PET image analysis have revealed that systemic administration of PHP.eB-CAG in C57BL/6 mice leads to substantial transgene expression in peripheral organs [40]. High levels of transduction were specifically observed in the liver and kidney within the initial 4–21 h post administration. Furthermore, a biodistribution analysis demonstrated that the presence of PHP.eB-CAG-mNeonGreen in the liver remained consistently high for up to three weeks following administration [41]. In light of these results, our findings highlight the potential of our MiniLAP2 design when packaged into the PHP.eB capsid. This design demonstrated a substantial reduction in off-target transgene expression in peripheral tissues compared with the native LAP2 promoter following systemic administration in C57BL/6 mice.

In this study, we aimed to achieve targeted transgene expression, specifically in brain neurons, by using a compact promoter sequence delivered via an rAAV vector. Our results revealed several noteworthy and biologically significant findings that hold promise for the development of novel promoters designed to overcome existing challenges. This study demonstrates the potential utility of MiniLAP2 in treating brain-related genetic disorders, as it possesses the advantage of being compatible with AAV vectors because of its compact size, and it leads to robust and sustained transgene expression in brain neurons while reducing off-target effects on peripheral tissues after intravenous administration.

## Materials and methods

### Construction and production of AAVs

All AAV vectors were produced by the Princeton Neuroscience Institute (PNI) Viral Core Facility (Princeton Neuroscience Institute, Princeton University), and contained mCherry driven by MiniLAP2 or LAP2. These vectors also include WPRE (woodchuck hepatitis virus post-transcriptional regulatory element) and human growth hormone polyadenylation (pA) sequence. The AAV plasmid was transfected into 293 T cells using PEI MAX (Polysciences, Warrington, PA, USA) and culture in six-well plates. Three days later, the mCherry-positive fluorescent cells were imaged with a Nikon Ti-E inverted epifluorescence microscope (Nikon Instruments, Tokyo, Japan). The resulting AAV plasmids were selected and packaged into ssAAV-PHP.eB serotype as previously described [33]. The AAV titer was measured by qPCR using TaqMan (Thermo Fisher Scientific, Rockford, IL, USA) and reported as genome copies (gc) per ml [42, 43].

### Animals

Five-week-old wild-type C57BL/6 male mice (Jackson Laboratories, Bar Harbor, ME, USA) were used for all experiments. The mice were maintained under a 12-hr light/dark cycle with ad libitum access to food and water. Special care was taken to minimize suffering and to reduce the number of animals used to the minimum required for statistical inference. All animal procedures were approved by the Institutional Animal Care and Use Committee of Princeton University (protocol 1947-19). Intravenous administration of rAAV vectors was performed via injection into the retro-orbital sinus. In this study, we used a cohort of 12 mice, which were distributed into three groups, each consisting of four mice. Specifically, one group received injections of PHP.eB-LAP2 (100 μl of 5 × 10^11^ gc), another received PHP.eB-MiniLAP2 (100 μl of 5 × 10^11^ gc) injections, and the third group served as a negative control and received buffer injections. Negative control mice were injected with 100 μl of buffer (1X PBS, 172 mM NaCl, 0.001% Pluronic F68, and 5% glycerol) without AAV. Animals were euthanized 30 days post-inoculation via intraperitoneal injection of ketamine (400 mg/kg)/xylazine (50 mg/kg), followed by perfusion with 4% paraformaldehyde (Fisher Scientific, Waltham, MA).

### Histology

Whole brain and spinal cord tissues were post-fixed for 2 h at room temperature (RT). The liver, kidneys, and quadriceps muscles were post-fixed for 24 h at 4 °C. All tissues were dehydrated in a sucrose gradient as previously described [44]. Paraffin blocks and sections (5 µm thickness) for immunohistochemistry (IHC), frozen blocks followed by sections (10 µm thickness) for and immunofluorescence (IF) and in situ hybridization (ISH) were prepared by HistoWiz, Inc. (Brooklyn, NY, USA).

### IHC and IF staining

Tissues to be evaluated by chromogenic IHC detection of mCherry were counterstained with hematoxylin. All staining procedures were performed using a Leica Bond RX Automated Stainer (Leica Microsystems, Wetzlar, Germany) by HistoWiz Inc. IF staining of brain and spinal cord sections was performed as previously described [33]. Briefly, cryosections were incubated with primary antibodies overnight at 4 °C, followed by incubation with the appropriate secondary antibodies for 1 h at RT (Supplementary Table 1). Nuclei were stained with 4’,6-diamidino-2-phenylindole (DAPI) (Thermo Fisher Scientific) and mounted with Vectashield® Vibrance antifade mounting media (Vector Laboratories, Newark, CA, USA).

### mRNA/DNA ISH

ISH detection of mCherry mRNA was performed using RNAscope® 2.5 High Definition (HD) Red assay (Advanced Cell Diagnostics [ACD], Newark, CA, USA). Briefly, brain sections were boiled for 15 min in the target retrieval solution and pretreated with proteinase plus solution for 30 min at 40 °C. Pretreated tissues were incubated with RNAscope® target probes (Supplementary Table 2) for 2 h at 40 °C and counterstained with hematoxylin. To detect mCherry DNA, the protocol was adapted according to the manufacturer’s recommendations and included incubation with RNase A (5 mg/ml, QIAGEN, Hilden, Germany) for 30 min at 40 °C immediately before hybridization with the DNAscope® target probe (Supplementary Table 2) via overnight incubation at 40 °C. The tissues were then counterstained with hematoxylin.

### Image analysis

Whole-slide scanning (40x) was performed using an Aperio AT2 scanner (Leica Microsystems). Morphometry of the resulting histological images was performed using HALO® software by HistoWiz Inc. Fluorescent images were captured using a Leica SP8-LSCM confocal microscope (20x). Whole brains were imaged using a NanoZoomer S60 microscopic scanner (Hamamatsu, Japan). Image reconstructions of z-stacks and intensity projection images were generated using ImageJ (https://imagej.nih.gov/ij/) and quantified using QuPath 0.3.0 software (https://qupath.github.io) [45]. The fluorescence intensity was calculated using an adaptation of the corrected total cell fluorescence formula [46]. Briefly, Cells were selected by drawing a region of interest (ROI) by tracking cells in DAPI expression and normalized to the background intensity from non-fluorescent cells. To RNA/DNA ISH the average number of dots per cell was calculated using the following ACD scoring criteria: 0 (no staining or <1 dot/10 cells), 1 (1–3 dots/cell), 2 (4–9 dots/cell and/or few to no dot clusters), 3 (10–15 dots/cell and/or <10% of the dots in clusters), and 4 (>15 dots/cell and/or >10% of the dots in clusters).

### Statistical analysis

No data was excluded from analysis. The allocation of animals and samples to separate groups was randomized. A two-tailed Student’s *t*-test was used to evaluate differences between the two groups. One-way analysis of variance (ANOVA) followed by Tukey’s multiple comparisons test was used to evaluate differences among multiple groups. Statistical significance was set at *P* < 0.05. Data are presented as mean ± standard error of the mean (SEM). Statistical analyses were performed using the GraphPad Prism 9.0 software (GraphPad, La Jolla, CA, USA).

### Supplementary information

Supplementary Figure 1

## Data Availability

The data presented in this study are available in “Engineered compact pan-neuronal promoter from Alphaherpesvirus LAP2 enhances target gene expression in the mouse brain and reduces tropism in the liver.” Original findings are available upon request.
