# Supplementary material for: Engineered compact pan-neuronal promoter from Alphaherpesvirus LAP2 enhances target gene expression in the mouse brain and reduces tropism in the liver
Source: Gene Ther. 2023 Nov 27;31(5-6):335–44. doi: 10.1038/s41434-023-00430-0 (PMC11090813; doi:10.1038/s41434-023-00430-0)
Supplement: Supplementary file 1 — Supplementary Figure 1 [file 41434_2023_430_MOESM1_ESM.docx]

**Supplementary Materials**

**Supplementary Figure 1**

**
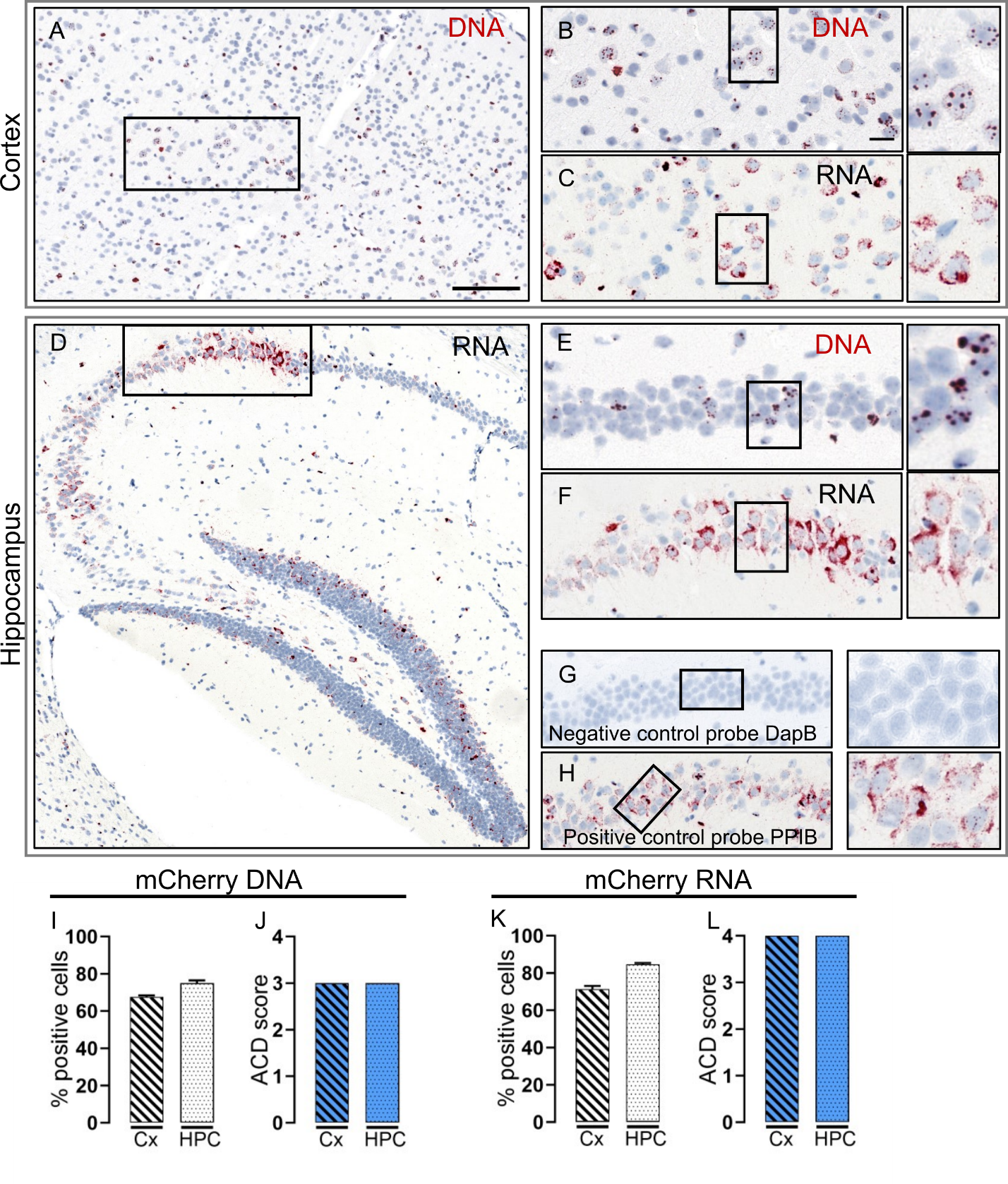
**

**Supplementary Figure 1.** Quantitative image-based data analysis of AAV genomes and transgene RNA detection in the brain. **(A, B, E)** mCherry DNA-and **(C, D, F)** mCherry RNA-positive cells were identified using an alkaline phosphatase-fast red detection method. Nuclei were counterstained with hematoxylin. In addition, images generated using **(G)** a negative control probe (bacterial dihydrodipicolinate reductase [DapB]) and (H) a positive control probe (Peptidylprolyl Isomerase B [PPIB]) targeting expression in HPC CA2. Transgene DNA was detected using a mCherry sense probe to target **(A, B)** the cortex (Cx) and **(E)** the hippocampus (HPC). Transgene RNA was identified using a mCherry antisense probe to target **(C)** the Cx and **(D, F)** the HPC. The inset squares show the fields shown at higher magnification (on the right); scale bars, 50 µm and 200 µm. (I) Percentage transgene DNA-positive and **(K)** transgene RNA-positive cells and **(J, L)** average number of dots per cell in the Cx and HPC sections, respectively. The ACD criteria for scoring dots per cell (0–4) are described in the Methods section.

**Supplementary Table 1.** Antibodies used for immunofluorescence and immunohistochemical staining.

| **Antibody Name** | **Dilution** | **Source** |
| --- | --- | --- |
| Rabbit anti-RFP | 1:500 | Thermo Fisher Scientific, Rockford, IL, USA |
| Chicken anti-mCherry | 1:250 | Abcam, Cambridge, MA, USA |
| Mouse anti-NeuN | 1:500 | Millipore Sigma, Temecula, CA, USA |
| Mouse anti-GFAP | 1:500 | Millipore Sigma, Temecula, CA, USA |
| Rabbit anti-Iba1 | 1:500 | Wako Pure Chemicals Industries, Richmond, VA, USA |
| Rabbit anti-Olig2 | 1:250 | Millipore Sigma, Temecula, CA, USA |
| Rabbit anti-GAD67 | 1:100 | Santa Cruz Biotechnology, Dallas, TX, USA |
| Chicken anti-TH | 1:300 | Aves Labs, Davis, CA, USA |
| Alexa Fluor 488-vGlut2 | 1:100 | Millipore Sigma, Temecula, CA, USA |
| Alexa Fluor 488 Donkey anti-Rabbit IgG | 1:1000 | Thermo Fisher Scientific, Rockford, IL, USA |
| Alexa Fluor 488 Donkey anti-Mouse IgG | 1:1000 | Thermo Fisher Scientific, Rockford, IL, USA |
| Alexa Fluor 647 Donkey anti-Rabbit IgG | 1:1000 | Thermo Fisher Scientific, Rockford, IL, USA |
| Alexa Fluor 647 Donkey anti-Chicken IgG | 1:500 | Thermo Fisher Scientific, Rockford, IL, USA |

**Supplementary Table 2**. RNAscope/BaseScope® probes were used in this study.

| **Probe Name** | **Description** | **Source** |
| --- | --- | --- |
| Probe-mCherry | Anti-sense probe targeting | ACD431201 |
| Positive control Probe-PPIB | Anti-sense probe targeting | ACD313911 |
| Negative control Probe-DapB | Anti-sense probe targeting | ACD310043 |
| Probe-mCherry-O5 | Sense probe targeting | ACD1107941-C1 |

ACD: Advanced Cell Diagnostics, Newark, CA, USA
